# Frog Skin-Derived Peptides Against *Corynebacterium jeikeium:* Correlation between Antibacterial and Cytotoxic Activities

**DOI:** 10.3390/antibiotics9080448

**Published:** 2020-07-26

**Authors:** Bruno Casciaro, Maria Rosa Loffredo, Floriana Cappiello, Walter Verrusio, Vito Domenico Corleto, Maria Luisa Mangoni

**Affiliations:** 1 Center For Life Nano Science@Sapienza, Istituto Italiano di Tecnologia, Viale Regina Elena 291, 00161 Rome, Italy; bruno.casciaro@iit.it; 2 Laboratory affiliated to Pasteur Italia-Fondazione Cenci Bolognetti, Department of Biochemical Sciences, Sapienza University of Rome, P.le Aldo Moro 5, 00185 Rome, Italy; mariarosa.loffredo@uniroma1.it (M.R.L.); floriana.cappiello@uniroma1.it (F.C.);; 3 Department of Clinical Internal, Anesthesiological and Cardiovascular Sciences, Sapienza University of Rome, 00185 Rome, Italy; walter.verrusio@uniroma1.it; 4 Digestive Endoscopy Unit, Department of Medical-Surgical Sciences and Translational Medicine, Sant’Andrea Hospital, Sapienza University of Rome, Via di Grottarossa, 1035, 00189 Rome, Italy; vito.corleto@uniroma1.it

**Keywords:** *Corynebacterium jeikeium*, antibiotic resistance, antimicrobial peptides, minimum inhibitory concentration, minimum bactericidal concentration, hemolysis, cytotoxicity, cosmetics

## Abstract

*Corynebacterium jeikeium* is a commensal bacterium that colonizes human skin, and it is part of the normal bacterial flora. In non-risk subjects, it can be the cause of bad body smell due to the generation of volatile odorous metabolites, especially in the wet parts of the body that this bacterium often colonizes (i.e., groin and axillary regions). Importantly, in the last few decades, there have been increasing cases of serious infections provoked by this bacterium, especially in immunocompromised or hospitalized patients who have undergone installation of prostheses or catheters. The ease in developing resistance to commonly-used antibiotics (i.e., glycopeptides) has made the search for new antimicrobial compounds of clinical importance. Here, for the first time, we characterize the antimicrobial activity of some selected frog skin-derived antimicrobial peptides (AMPs) against *C. jeikeium* by determining their minimum inhibitory and bactericidal concentrations (MIC and MBC) by a microdilution method. The results highlight esculentin-1b(1-18) [Esc(1-18)] and esculentin-1a(1-21) [Esc(1-21)] as the most active AMPs with MIC and MBC of 4–8 and 0.125–0.25 µM, respectively, along with a non-toxic profile after a short- and long-term (40 min and 24 h) treatment of mammalian cells. Overall, these findings indicate the high potentiality of Esc(1-18) and Esc(1-21) as (i) alternative antimicrobials against *C. jeikeium* infections and/or as (ii) additives in cosmetic products (creams, deodorants) to reduce the production of bad body odor.

## 1. Introduction

Corynebacteria are Gram-positive, aerobic, catalase-positive, generally non-motile rods, and this genus comprises *Corynebacterium diphtheriae* and other different species defined as nondiphtheriae corynebacteria (diphtheroids) [1,2]. *Corynebacterium jeikeium* (commonly known as group JK corynebacterium by the Centers for Disease Control and Prevention) is part of the human skin flora as *Staphylococcus epidermidis* and is among the most common bacteria isolated from hospitalized patients [3,4,5]. In particular, its colonization affects groin and axilla where the moist environment favors the formation of malodor substrates leading, in some cases, to an unpleasant body smell [6,7]. *C. jeikeium* is ubiquitous and largely innocuous, and importantly, it offers substantial epidermal protection thanks to the production of bacteriocin-like compounds that are used to counteract possible pathogenic competitors [8]. However, in the last few decades, *C. jeikeium* has become the etiological agent of infections associated with skin wounds and implanted medical devices, as well as of nosocomial infections especially in hospitalized patients [1]. Indeed, in these latter, due to the impaired immune system, “helpful” microorganisms belonging to the commensal flora and regularly living within the host can turn into opportunistic pathogens leading to the appearance of infections with an illness state [9,10,11,12,13]. Considering the rising enhancement of people recovering from chemotherapy treatments or with a compromised defense mechanism, the number of these nosocomial infections (encompassing endocarditis, pneumoniae, peritonitis, and enteritis) is actually extremely alarming [14,15]. The first line therapies to combat *C. jeikeium-*associated infections include glycopeptides vancomycin or teicoplanin. Unfortunately, the inappropriate usage of these antibiotics and the high selective pressure have made this bacterium resistant to vancomycin, teicoplanin, linezolid, quinupristin-dalfopristin, daptomycin, and tigecycline [16]. Hence, new agents with antimicrobial activity are urgently needed. Naturally-occurring antimicrobial peptides (AMPs) represent a valid alternative to traditional antibiotics, as they are active against a broad spectrum of microbes, from Gram-positive and Gram-negative bacteria to fungi and viruses [17,18,19,20,21,22,23]. Furthermore, compared to conventional drugs, they have further biological functions, such as a wound-healing, antidiabetic, anti-inflammatory, and immune-modulatory activities [24,25,26,27,28,29,30,31]. Amphibian skin secretion is considered a rich source of broad-spectrum AMPs, and over the years, numerous peptides have been isolated and classified into the corresponding families, such as esculentins, temporins, and bombinins H [32,33]. Recently, we characterized the potent effectiveness of two derivatives of the N-terminal part of two frog skin AMPs, i.e., esculentin-1a and -1b, namely esculentin-1a(1-21) (Esc(1-21)) and esculentin-1b(1-18) (Esc(1-18)), respectively, especially against alarming human pathogens, such as *Pseudomonas aeruginosa* and *Candida albicans,* either *in vitro* or *in vivo* [34,35,36]. Here, for the first time, we explore the efficacy of these two alpha-helical peptides against *C. jeikeium* by determining their minimum inhibitory concentrations (MIC) and compare this activity with that of other peptide isoforms belonging to different AMP families (i.e., the alpha-helical temporin A, temporin B, temporin G, and bombinin H_2_). The minimum bactericidal concentration (MBC) of both esculentin derivatives is also determined, along with mechanistic studies to gain insight into their mode of action. Moreover, to evaluate the safety profile of these peptides for the development of new antimicrobials, their hemolytic activity on mammalian erythrocytes and their effect on the viability of three different mammalian cell lines are also investigated.

## 2. Results

### 2.1. Antimicrobial Activity of Frog Skin-Derived AMPs

**Figure 1:**
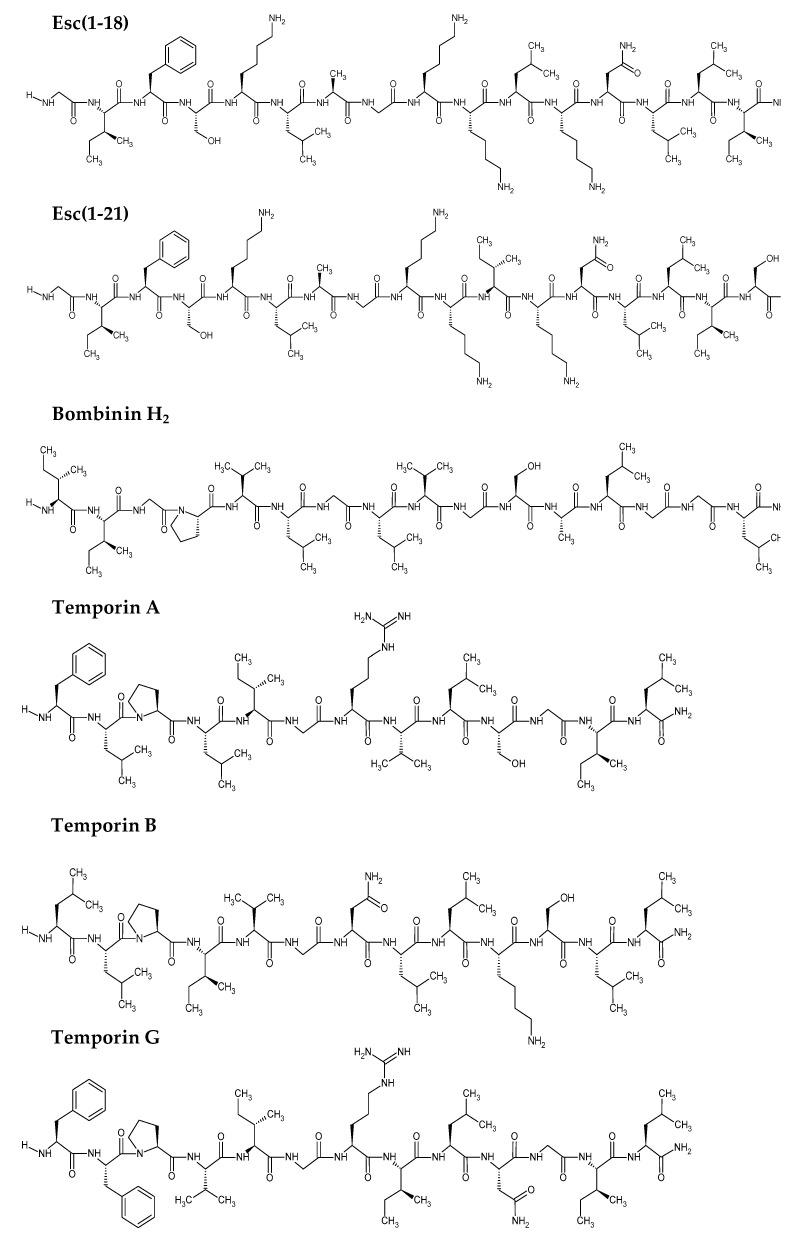
Names and primary structures of the selected peptides. Chemical structures were drawn with ChemSketch, Advanced Chemistry Development, Inc. (ACD/Labs).

Esc(1-18) and Esc(1-21), collectively named esculentin peptides, as well as the frog skin-derived AMPs temporin A, temporin B, temporin G, and bombinin H_2_ (Figure 1) were tested against the reference strain *C. jeikeium* ATCC BAA-949 to assess the MIC, i.e., the lowest peptide concentration able to visually inhibit microbial growth after 20 h of incubation at 37 °C.

As reported in Table 1, esculentin peptides showed the strongest efficacy in inhibiting *C. jeikeium* growth with MICs of 4 µM and 0.125 µM for Esc(1-18) and Esc(1-21), respectively. This result was quite surprising, considering that esculentin peptides have a well-documented preferential activity towards Gram-negative bacteria [36,37]. In comparison, MICs ranging from 8 to 16 µM were obtained for the other selected AMPs. Unlike temporins and bombinin H_2_, esculentin peptides are lysine-rich AMPs with a higher cationicity and lower hydrophobicity, as indicated by the corresponding grand average of hydropathicity index (GRAVY; Table 1), which is used to represent the hydrophobicity value of a peptide [38].

Interestingly, when the most active esculentin peptides were tested for their bactericidal activity, they were found to cause almost the complete killing of the bacterial population with an MBC (i.e., the lowest peptide concentration able to cause a ≥3 log reduction in the number of bacterial cells) 2-fold higher than the corresponding MIC, i.e., 8 µM and 0.25 µM for Esc(1-18) and Esc(1-21), respectively.

### 2.2. Membrane Permeabilization

**Figure 2:**
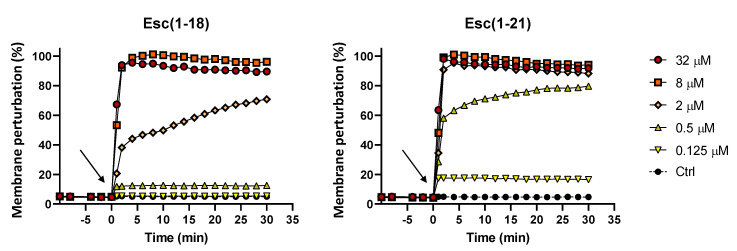
Membrane perturbation assay performed with the Sytox Green dye. The percentage of membrane damage was calculated with respect to the maximum membrane permeabilization obtained with the highest peptide concentration (32 µM) and the addition of 1 mM EDTA + 0.5% Triton X-100. Arrows indicate the addition of the peptide. Data points are the mean of triplicate measurements from a single experiment representative of three independent experiments. Controls (Ctrl) are cells not treated with the peptides.

Esculentin peptides are known to display an antibacterial activity mainly through a membrane-perturbing mechanism of action. To verify their ability to perturb the cytoplasmic membrane of Gram-positive bacteria, such as *C. jeikeium*, a Sytox Green assay was performed. Sytox Green is a fluorescent probe unable to cross intact membranes, and its fluorescence intensity significantly increases upon binding to nucleic acids. As shown in Figure 2, the rapid increase of the fluorescent signal immediately after peptide addition to the bacterial cells (arrow) indicated that the perturbation of the membrane was the result of the peptide-induced membrane damage, allowing the internalization of the probe with a consequent binding to the bacterial DNA. Both esculentin AMPs manifested a dose-dependent fast kinetic membrane destabilization with a total perturbation of the phospholipid bilayer already within the first minutes of treatment at the highest concentration of 32 µM. According to the MIC and MBC values, the most active Esc(1-21) exhibited a stronger effect compared to Esc(1-18), even at a concentration as low as 0.5 µM with about 60% membrane damage within the first 2 min.

### 2.3. Cytotoxicity

#### 2.3.1. Hemolytic Activity

**Figure 3:**
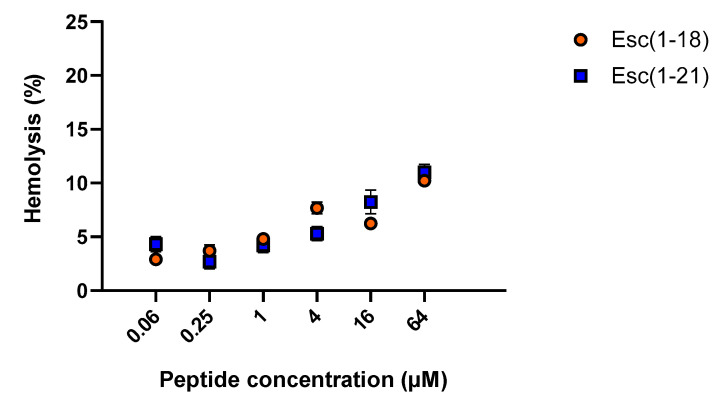
Effect of Esc(1-18) and Esc(1-21) on mammalian red blood cells after 40 min of incubation at 37 °C. The percentage of hemolysis was calculated with respect to the control (cells treated with vehicle). Data are the means ± standard error of the mean (SEM) of three independent experiments.

To evaluate the potential cytotoxicity of esculentin peptides in the short term, they were tested for the ability to lyse mammalian red blood cells after 40 min of treatment at 37 °C. Both AMPs caused a weak and similar hemolysis with an ~10% release of hemoglobin at a concentration of 64 µM, which is significantly higher than the antimicrobial dosages, thus suggesting their safety profile for short-term treatment (Figure 3).

#### 2.3.2. Peptides’ Effect on the Metabolic Activity of Mammalian Cells

**Figure 4:**
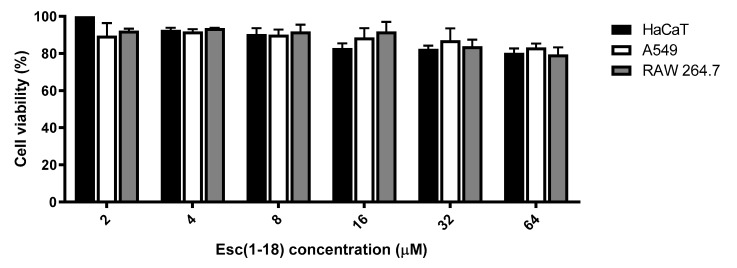
Dose-dependent effect of Esc(1-18) at different concentrations on the viability of HaCaT, A549, and RAW 264.7 cell lines after 24 h of treatment. The percentage of metabolically-active cells compared to untreated control samples is reported on the Y-axis. All data are the mean of three independent experiments ± SEM.

Since the long-term cytotoxicity of Esc(1-21) was extensively described in earlier studies [40,41], we similarly investigated the potential cytotoxic effect of Esc(1-18) after a long time interval (24 h) against three different mammalian cell lines, i.e., the human immortalized keratinocytes (HaCaT cells), the human alveolar epithelial A549 cells, and the murine RAW 264.7 macrophages, by the 3-(4,5-dimethylthiazol-2-yl)-2,5-diphenyltetrazolium bromide (MTT) assay (see Materials and Methods). Figure 4 shows the percentage of cell viability after 24 h of exposure to Esc(1-18) in the concentration range of 2–64 µM. Even at the highest peptide concentration tested, only a weak reduction in the percentage of metabolically-active cells was observed (about 20%) compared to the untreated control samples. Similar data were previously collected for viability assays performed with Esc(1-21) at the same concentrations, except for the greater cytotoxicity against macrophages at 64 µM (i.e., about 50% cell viability) [41]. Lacking the harmful effect of esculentin peptides, particularly for Esc(1-18) at the antimicrobial concentrations (MIC and MBC values), suggested they are safe compounds, also for long-term treatment.

## 3. Discussion

Having been considered harmless and protective to humans, *C. jeikeium* has turned into a relevant etiological agent of dangerous infections, especially in hospitalized people or patients under chemotherapy. These include skin and wound infections, meningitis, enteritis, osteomyelitis, pyelonephritis, prosthetic joint infections, peritonitis, pneumonia, and liver abscess in patients with AIDS [42,43,44,45,46,47,48,49,50]. Unfortunately, current antibiotic therapies are often ineffective due to the onset of antibiotic resistance [51,52]. Interestingly, AMPs are promising hits to open the door for the generation of a new class of antimicrobials. In recent years, we thoroughly investigated the activity of Esc(1-18) and Esc(1-21) against a large number of microorganisms. However, no studies have been conducted so far for these peptides, as well as for other AMPs, against *C. jeikeium*. Here, for the first time, we analyzed the effect of some frog skin AMPs against this bacterium and selected the esculentin peptides as the most active molecules with an MIC of 0.125 and 4 µM for Esc(1-21) and Esc(1-18), respectively. This outcome is in sharp contrast with the weaker activity of Esc(1-21) previously recorded against other Gram-positive bacteria (e.g., *Staphylococcus aureus*, *S. epidermidis*), where MICs ranging from 1 to 64 µM were found [37]. Note that both the higher cationicity and lower hydrophobicity of esculentin peptides compared to the other AMPs used for comparison (Table 1) would promote the peptide interaction with the anionic phospholipid headgroups of the bacterial membrane, thus explaining the higher antimicrobial activity of esculentin peptides. However, we cannot exclude the contribution of other chemical/physical features of the peptides (i.e., amphipathicity, length, oligomeric state) for the final outcome. Analogously, among esculentin peptides, the significantly higher antibacterial activity of Esc(1-21) compared to the shorter analog Esc(1-18) is likely due to the higher net positive charge and longer size of the former. Indeed, a minimum length for a peptide in alpha-helical conformation to span and perturb a phospholipid bilayer (∼30 Å thick) is 20 amino acid*s.* Both esculentin peptides displayed the capability to kill *C. jeikeium* at a concentration two-fold higher than the MIC. Remarkably, despite the obtained MICs being comparable to those of traditional antibiotics [53,54], we have to take into account the additional advantageous features owned by these AMPs. As an example, we already demonstrated the ability of Esc(1-21) to promote re-epithelialization of a pseudo-wound by stimulating the migration of human keratinocytes, a relevant property for the therapeutic development of these peptides. Indeed, besides contributing to the elimination of *C. jeikeium* skin/wound-associated infections, the peptide would help to repair the damaged epithelial tissue. In addition, the membrane-perturbing activity makes these AMPs even more interesting compounds, as bacteria are less prone to develop resistance to them. This is because the acquisition of resistance to AMPs would imply a complete reorganization of the bacterial membrane; an energetically unfavorable process not compatible with bacterial survival [55,56,57,58,59,60]. Moreover, in contrast with mammalian AMPs, esculentin peptides can preserve antimicrobial activity in the presence of biological fluids [37]. Esc(1-21) was found to induce a complete permeabilization of the bacterial membrane at a concentration range from 2 to 32 µM. Differently, only 50% of the membrane perturbation had been previously achieved by this peptide against other Gram-positive bacteria (i.e., *Streptococcus agalactiae* ATCC 13813) at the higher concentration of 50 µM [36]. Finally, both esculentin peptides displayed a non-toxic profile on different types of mammalian cells, e.g., erythrocytes, keratinocytes, alveolar epithelial cells, and macrophages, either after a short- or long-term treatment. Importantly, HaCaT cells are a reliable and helpful *in vitro* model to determine the toxicity of various agents on the skin layer because keratinocytes represent 95% of the epidermal cells [61,62]. In comparison, the evaluation of the peptides’ effect against lung epithelial cells, i.e., the A549 alveolar cell line, and immune cells, like macrophages, is of great interest for their crucial role in orchestrating both immune defense and inflammatory responses [63,64,65]. It is noteworthy that esculentin peptides may be developed not only as alternative antimicrobials against *C. jeikeium* infections, but also as additives in cosmetic products (e.g., creams, deodorants) aimed at countering the colonization of *C. jeikeium* and, as a result, axillary malodor formation. In fact, this bacterium is involved in the generation of volatile odorous metabolites, attributed to the bacterial degradation of skin lipids and specific odor precursors that are responsible for the unpleasant human body smell, in sweat secretions [6,7,66]. Frequent showers and different soaps cannot solve the problem of the so-called “wild axilla”, thus provoking serious psychological concerns. Patients suffering from bad smell can adopt various atypical behaviors minimizing social interactions. This can lead to anxiety, decreased self-esteem, and low quality of life due to social difficulties, e.g., avoiding intimacy [67,68]. Overall, the potent antimicrobial action of esculentin peptides, especially of Esc(1-21) [MIC, 0.125 µM], and their safety profile make them attractive molecules for therapeutic and/or cosmetic application. Based on the amino acid sequence analysis conducted through bioinformatic platforms (https://webs.iiitd.edu.in/raghava/algpred/submission.html and https://web.expasy.org/protparam, [39,69]), it has been predicted that esculentin peptides are devoid of allergenic properties and have an estimated half-life time in mammalian reticulocytes (*in vitro*) of about 30 h, which is a compatible time for possible daily usage. This is also consistent with our recent *in vivo* efficacy studies showing: (i) an antimicrobial efficacy of Esc(1-21), 36 h after intra-tracheal administration in murine models of acute bacterial lung infection; and (ii) the absence of immunogenicity in mice [70,71]. In the work of Rahnamaeian and Vilcinskas, the authors already emphasized the feasibility of short-sized AMPs as cosmetic ingredients of topical formulations such as creams, lotions, shampoos, and wound dressings to deter dermatological pathogens [72]. As proof of this, Haisma and coworkers designed cream/gel formulations (e.g., a water-in-oil cream with lanolin, an oil-in-water cream with polyethylene glycol hexadecyl ether, and a hypromellose gel) containing the LL-37-derived AMP P60.4Ac to successfully eradicate methicillin-resistant *S. aureus* from colonized human epidermal models [73]. Taken all together, the data presented in this work demonstrated for the first time the high potentiality of esculentin peptides as a new choice to fight the undesirable infections caused by *C. jeikeium* in both healthy and susceptible individuals.

## 4. Materials and Methods 

### 4.1. Microorganism and Cell Lines

The microorganism used in the study was the reference strain *C. jeikeium* ATCC BAA-949. The culture media used for bacterial growth and the various assays were tryptone soy broth and agar (TSB and TSA, respectively) supplemented with 0.1% Tween 80 (TSB^+^ and TSA^+^). Furthermore, the following cell cultures were employed: the human immortalized keratinocyte cell line, HaCaT (from AddexBio, San Diego, CA, USA), the human type II alveolar epithelial cell line A549, and the murine RAW 264.7 macrophage cell line (from the American Type Culture Collection, Manassas, Va). The three cell lines were cultured in Dulbecco’s Modified Eagle’s Medium supplemented with 4 mM glutamine (for HaCaT cells) or 2 mM glutamine (for the A549 and RAW 264.7 cell lines), 10% heat-inactivated fetal bovine serum (FBS), and 0.1 mg/mL of penicillin and streptomycin at 37 °C and 5% CO_2_, in 25 cm^2^ or 75 cm^2^ flasks. In the case of macrophages, sodium pyruvate and non-essential amino acids were also added to the culture medium.

### 4.2. Peptides

Esc(1-18), Esc(1-21), temporin A, temporin B, temporin G, and bombinin H_2_ were purchased from Biomatik (USA/Canada). Each peptide was assembled by stepwise solid-phase synthesis and purified via reverse-phase high-performance liquid chromatography to a purity of 98% using a gradient of acetonitrile in 0.1% aqueous trifluoroacetic acid (from 28% to 100% in 30 min) at a flow rate of 1.0 mL/min. Molecular masses were verified by electron spray ionization mass spectrometry.

### 4.3. Antimicrobial Assays

The MIC of the different AMPs against *C. jeikeium* ATCC BAA-949 were determined following the previously described procedure with some modifications [57]. The bacterium was grown in TSB^+^ up to an optical density (O.D.) of 0.8 (λ = 590 nm) and diluted to reach a concentration of 2× 10^6^ colony forming units (CFU) per mL. Aliquots (50 µL) of this dilution were added to 50 µL of TSB^+^ supplemented with 2-fold serial dilution of peptides previously dispensed in the wells of a 96 well plate. Controls were bacteria not treated with the peptides. The plate was then incubated for 20 h at 37 °C, and the MIC was defined as the lowest peptide concentration that visually inhibits microbial growth (absence of turbidity) after 20 h incubation. For determining the MBC of the most potent peptides, i.e., Esc(1-18) and Esc(1-21), aliquots from MIC, 2× MIC and 4× MIC wells were spread onto TSA^+^ plates for CFU counting after an overnight incubation. MBC was defined as the lowest peptide concentration able to cause a ≥3 log reduction in the number of cells of the initial inoculum after 20 h of incubation.

### 4.4. Membrane Permeabilization: Sytox Green Assay

To assess the ability of Esc(1-21) and Esc(1-18) to perturb the cytoplasmic membrane permeability of *C. jeikeium* ATCC BAA-949, the Sytox Green assay was performed as previously reported, with some modifications [35]. Approximately 1× 10^7^ CFU/mL were incubated with 1 μM Sytox Green in PBS for 5 min in the dark. After peptide addition, changes in fluorescence intensity (λ exc = 485 nm, λ ems = 535 nm) caused by the binding of the dye to intracellular DNA were monitored for 30 min in the microplate reader (Infinite M200, Tecan, Salzburg, Austria) at 37 °C and plotted as the percentage of membrane perturbation relative to that obtained after treating bacteria with the highest peptide concentration (32 μM) and the addition of 1 mM EDTA + 0.5% Triton X-100 (final concentration). The peptide concentrations ranged from 0.125 to 32 μM. Controls were cells not treated with the peptides.

### 4.5. Hemolytic Assay

The short-term cytotoxicity of Esc(1-18) and Esc(1-21) was evaluated against sheep red blood cells (OXOID, SR0051D) by adapting the already described procedure [74]. Erythrocytes (O.D. of 0.5 at λ= 500 nm) in 0.9 % (w/v) NaCl were incubated for 40 min at 37 °C with 4-fold serial dilutions of Esc(1-18) and Esc(1-21) (0.06–64 μM). Complete lysis was obtained by resuspending erythrocytes in distilled water. All samples were centrifuged for 5 min at 900× *g*, and the amount of hemoglobin released in the supernatant by lysed red blood cells was measured at 415 nm using a microplate reader (Infinite M200; Tecan, Salzburg, Austria). 

### 4.6. Cytotoxicity Test on Mammalian Cell Lines 

The long-term cytotoxicity of Esc(1-18) was investigated by the MTT assay according to [41], as previously carried out for Esc(1-21). MTT (Sigma-Aldrich, St. Luis, MO, USA) is a yellow dye that, upon intracellular entry, is converted into insoluble and dark purple formazan crystals by mitochondrial dehydrogenases. This reduction occurs only in metabolically-active cells; therefore, the colorimetric absorbance is directly proportional to the cell viability. About 4 × 10^4^ cells, suspended in DMEM supplemented with glutamine (at the concentration indicated for each cell line) and 2% FBS without antibiotic, were plated in triplicate wells of a microtiter plate. After overnight incubation at 37 °C and 5% CO_2_, the medium was replaced by fresh serum-free medium containing the peptide at different concentrations. Cells not treated with the peptide were used as controls. After 24 h of incubation at 37 °C in a 5% CO_2_ atmosphere, the medium was removed, and MTT solution in Hank’s buffer (final concentration 0.5 mg/mL) was added to each well. The plate was incubated for 4 h at 37 °C and 5% CO_2_. Afterwards, the formazan crystals were dissolved by adding acidified isopropanol, and the absorbance of each well was measured at 570 nm using the microplate reader (Infinite M200; Tecan, Salzburg, Austria) (cell viability was calculated by assuming a percentage of 100% for control cells without any peptide treatment).

### 4.7. Statistical Analysis 

Unless otherwise specified, all experiments were performed three times, and the obtained values were reported as the mean ± SEM.

## 5. Conclusions

AMPs are an effective alternative to conventional antibiotics in the battle to defeat microbial pathogens, due to their membrane-active microbicidal activity and to further biological properties. In the last few decades, *C. jeikeium* raised concerns in the clinical field because of the increased occurrence of its infections, especially in immunocompromised or hospitalized subjects. In non-risk patients, this bacterium normally colonizes skin (e.g., axilla), causing a bad smell. Here, for the first time, we characterized the efficacy of two derivatives of esculentin-1 peptides, i.e., Esc(1-18) and Esc(1-21), against *C. jeikeium* and highlighted their high potentiality as new antimicrobials with negligible cytotoxicity. Beyond the clinical relevance that these peptides can have in the scenario of antibiotic resistance, they represent excellent candidates to be used at low concentrations in the production of cosmetics designed to reduce bad body odors.

## Figures and Tables

**Table 1:** Antimicrobial activity (MIC) of different frog skin AMPs against *C. jeikeium*. The net charge at neutral pH and grand average of hydropathicity (GRAVY) of each peptide are also indicated. GRAVY values were provided by https://web.expasy.org [39].

Peptide	Sequence	MIC (µM)	Net Charge	GRAVY
Esc(1-18)	GIFSKLAGKKLKNLLISG-NH_2_	4	+5	0.383
Esc(1-21)	GIFSKLAGKKIKNLLISGLKG-NH_2_	0.125	+6	0.338
Temporin A	FLPLIGRVLSGIL-NH_2_	8	+2	1.808
Temporin B	LLPIVGNLLKSLL-NH_2_	8	+2	1.638
Temporin G	FFPVIGRILNGIL-NH_2_	16	+2	1.577
Bombinin H_2_	IIGPVLGLVGSALGGLLKKI-NH_2_	8	+3	1.525
